# Dynamics of Visual Perceptual Echoes Following Short-Term Visual Deprivation

**DOI:** 10.1093/texcom/tgaa012

**Published:** 2020-04-13

**Authors:** Jakob C B Schwenk, Rufin VanRullen, Frank Bremmer

**Affiliations:** 1 Department of Neurophysics, University of Marburg, Marburg 35043, Germany; 2 Center for Mind, Brain and Behavior—CMBB, University of Marburg and Justus-Liebig-University Gießen, Marburg 35032, Germany; 3 Centre de Recherche Cerveau et Cognition, CNRS UMR 5549, Université de Toulouse, Toulouse 31052, France

**Keywords:** alpha, EEG, perceptual echoes, rhythmic sampling, short-term visual deprivation

## Abstract

The visual impulse-response function to random input as measured by EEG is dominated by the perceptual echo, a reverberation of stimulus information in the alpha range believed to represent active rhythmic sampling. How this response is generated on a cortical level is unknown. To characterize the underlying mechanisms, we investigated the echoes’ dynamics following short-term visual deprivation, which is known to modify the excitation/inhibition balance in visual cortex. We subjected observers to 150 min of light deprivation (LD) and monocular contrast deprivation (MD). Perceptual echoes were measured by binocular and dichoptic stimulation, respectively, and compared with a baseline condition. Our results show that the echo response is enhanced after LD, but not affected in temporal frequency or spatial propagation. Consistent with previous studies, MD shifted early response (0–150 ms) amplitudes in favor of the deprived eye, but had no systematic effect on the echoes. Our findings demonstrate that the echoes’ synchrony scales with cortical excitability, adding to previous evidence that they represent active visual processing. Their insensitivity to modulation at the monocular level suggests they are generated by a larger region of visual cortex. Our study provides further insight into how mechanisms of rhythmic sampling are implemented in the visual system.

## Introduction

Traditional approaches have characterized the visual response in the electroencephalogram (EEG) as the response to presentations of transient stimuli (event-related potentials, ERPs). Yet, natural visual information is continuously changing. This poses the question how the visual system achieves a temporal organization from that continuous input. Recently, VanRullen and MacDonald (2012) measured responses to random luminance sequences to characterize the visual impulse-response function (IRF) to continuous stimulation. Their results revealed a large alpha oscillation that begins after the ERP-like early response and is sustained for lags up to >1 s. This component, the perceptual echo, represents the rhythmicity that the visual system naturally selects from the random sequence. Recent studies have demonstrated that the echo response may be employed as a mechanism of active rhythmic sampling that can be modulated according to current visual demand (Gulbinaite et al. 2017; Benedetto et al. 2018). The organization of the evoked activity into cycles at the echo rhythm may be used to discretize the input, aiding further processing (Gaillard et al. 2020; for a detailed discussion, see also VanRullen 2016).

However, little is known about the neural underpinnings of the echo response. Generally, alpha oscillations are believed to be generated by local inhibitory feedback loops within the cortex (Lopes Da Silva et al. 1973; Lagerlund and Sharbrough 1989). High correlations between peak alpha and echo frequencies suggest that the latter is generated by the same oscillators. Yet, its behavior deviates from that traditionally found for alpha. For instance, attention leads to alpha desynchronization (Doppelmayr et al. 1998; Sauseng et al. 2005), whereas it increases echo amplitude (VanRullen and MacDonald 2012). Furthermore, the echo is strongly modulated by ambient luminance, whereas spontaneous alpha is not (Benedetto et al. 2018). Thus, to generate the echo, the visual system seems to engage the ongoing alpha rhythm in a unique way.

One approach to identify the neural mechanisms behind the echoes is to study their dynamics following disruptions in the cortical excitation/inhibition balance. Multimodal evidence suggests that alpha oscillations are closely associated with inhibition; alpha amplitude predicts transcranial magnetic stimulation (TMS) phosphene-thresholds between and within subjects (Romei et al. 2008a, 2008b) and is linked to inhibition of visual input in different tasks (Jensen 2002; Klimesch et al. 2007; Sauseng et al. 2005, 2009; Foxe and Snyder 2011). Peak alpha frequency shows small shifts with cortical activation level, with higher frequencies as a result of increased inhibitory feedback gain (Hindriks and van Putten 2013; Mierau et al. 2017). Finally, alpha oscillations are also directly affected by pharmacological disruptions to GABAergic neurotransmission (Ahveninen et al. 2007; Lozano-Soldevilla et al. 2014). Based on this association, we investigated, here, how the echoes are modulated by cortical excitability, to eventually identify the neural mechanisms that generate them.

An established paradigm to induce shifts in neural excitability is that of short-term visual deprivation. The visual system reacts to temporary loss of input by increasing excitability to maintain homeostatic balance of neural activity. In human studies, two commonly used paradigms are light deprivation (LD) and monocular deprivation (MD). Short-term LD (45–180 min) consistently increases excitability of the visual cortex, as measured by TMS phosphene-thresholds, and increases BOLD responses in V1 (Boroojerdi et al. 2000; Fierro et al. 2005; Pitskel et al. 2007). These changes are likely mediated by a down-regulation of GABAergic inhibition, thereby increasing firing rates of pyramidal cortical neurons (Boroojerdi et al. 2001; Huang et al. 2015). In MD, the loss of sensory information is limited to one eye. In a series of studies using contrast deprivation, Lunghi et al. demonstrated that after MD (120–150 min) the balance in binocular rivalry and early visual responses in V1 is shifted in favor of the deprived eye (Lunghi et al. 2011, 2015a; Binda et al. 2018). Importantly, they also found reduced GABA levels in V1 after MD (Lunghi et al. 2015b).

In summary, short-term LD and MD present reliable paradigms to induce states of increased excitability, affecting V1 globally and at the monocular level, respectively. Here, we investigated the effects of both types of deprivation on perceptual echoes. In experiment 1, we recorded echoes from binocular stimulation before and after the LD. In experiment 2, we examined monocular effects of MD on echoes measured separately for each eye through dichoptic stimulation.

## Materials and Methods

### Participants

Twenty-seven observers (16 female, age 19–33 years) participated in the two experiments (12 in experiment 1 and 16 in experiment 2; one observer took part in both experiments), all with normal or corrected-to-normal vision and no known neurological disorders. In experiment 2, eye dominance was assessed before the start of testing using the standard Porta eye dominance test. All experiments were approved by the local Ethics Committee (Psychology Department, University of Marburg), and the observers gave written informed consent prior to the start of testing in accordance with the Declaration of Helsinki.

### Stimuli and Task

We used the white-noise (WN) stimulation paradigm developed by VanRullen and MacDonald (2012) to measure visual IRFs in both experiments. The observer started each trial by a button press. After a variable delay period (300–500 ms), either one (experiment 1) or two (experiment 2) circular patches appeared on a black background while the observer fixated at the center of the screen. The luminance of each patch followed a random WN sequence between black (~0.1 cd/m^2^) and white (100 cd/m^2^) of 6.25 s duration. The sequences were unique and independent for each trial and individual patch and were updated on every frame at the respective monitor refresh rate (120 fps in experiment 1 and 240 fps in experiment 2). The observer’s task was to covertly monitor the stimulus patch for the appearance of a square that appeared inside the patch for 1 s on a random 25% of the trials (cf., VanRullen and MacDonald 2012). Observers gave their response by button press after the trial was complete. In experiment 2, the target appeared on only one side (left or right patch) chosen randomly for each appearance. The square followed the same luminance sequence with a certain offset controlling task difficulty. This offset was varied adaptively in a staircase procedure to keep the observer’s detection rate fixed at ~84%.

Stimulus presentation in both experiments was controlled by a Linux computer, using MATLAB version 9.6 (The MathWorks) and the Psychophysics Toolbox (Brainard 1997).

### Experimental Design

Our aim was to characterize the effects of two different deprivation regimes on visual IRFs. To this end, we subjected observers to WN stimulation in two experimental sessions: a baseline session (control) and one after 150 min of deprivation (deprivation) (the different deprivation procedures are described below). Due to the substantial total duration of the experiment, the two sessions were conducted on different days in random order across participants. To exclude possible effects of circadian rhythmicity, we confirmed that none of the observed effects correlated with the difference in time of day between sessions (signed difference, deprivation minus control: 54.0 ± 170.587 min and 140.687 ± 136.481 min (mean ± 1 SD) for experiments 1 and 2, respectively).

**Figure 1:**
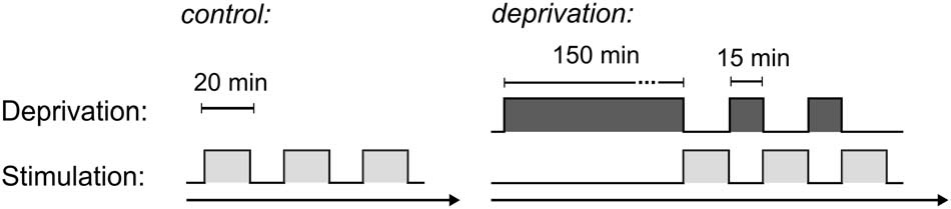
Sequence of experimental blocks in control and deprivation sessions. Stimulation blocks were fixed at a 20-min duration, during which observers proceeded with the trials at their own pace. In the deprivation session, stimulation immediately followed the offset of deprivation. The sessions were conducted on two separate days.

The stimulation sequences within each session are illustrated in Fig. 1. Both sessions consisted of three stimulation blocks. Previous studies have reported effects of short-term deprivation lasting ~15–20 min (Lunghi et al. 2011). Accordingly, we fixed the duration of stimulation blocks at 20 min. For the duration of these blocks, observers continued with the trial procedure at their own pace and were instructed to take breaks between trials when necessary. After 20 min, stimulation stopped at the next intertrial interval. This yielded a total number of 313.05 ± 6.17 trials per session (mean ± 1 SEM across both experiments). We did not balance the number of trials between sessions for the analyses reported here, except for one subject in experiment 1 that showed a large discrepancy in the number of trials. For all between-session effects, we confirmed that the effect was also present with trial numbers balanced.

In the deprivation session*,* the first block was preceded by a 150-min period of deprivation. The observers spent this time in the laboratory either with both eyes covered or one eye occluded (experiments 1 and 2, respectively), pursuing their own activities. An experimenter was present for the full time of deprivation in both experiments.

The three blocks were interleaved with breaks of 15 min. Observers spent this time in the laboratory with room lights on. Importantly, in the deprivation session, deprivation was resumed during this time, using the same procedure as in the main deprivation block.

### Experiment 1: LD

In the first experiment, we compared binocular IRFs to WN stimulation before and after LD. To this end, observers were presented with one circular stimulus patch (5.5° in diameter) per trial, located at 7° in the upper hemifield on the vertical meridian. Stimuli were presented on a ViewPixx 3D Lite LCD with a resolution of 1920 × 1080 pixel and a refresh rate of 120 fps. The observers sat in a dark room with their head on a chinrest positioned at 68 cm from the screen.

**Figure 2:**
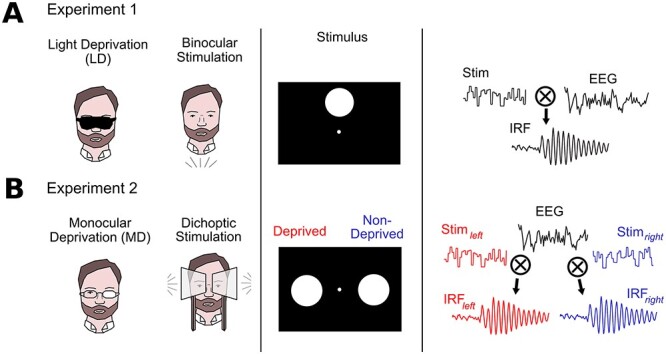
Summary of the paradigms used to measure impulse-response functions (IRFs) in the two experiments. The WN stimulation and cross-correlation procedure are the same as in VanRullen and MacDonald (2012). (*A*) Experiment 1: For the LD, observers wore a blindfold covering both eyes. Stimulation consisted of a single patch viewed binocularly, resulting in a single, binocular IRF. (*B*) Experiment 2: Observers wore a translucent patch over one eye (MD). Stimulation consisted of two patches presented each to one eye only (in the ipsilateral hemifield). From the recorded EEG, IRFs were then computed for deprived and nondeprived eye separately. The side of deprivation was pseudo-randomized among observers.

During the period of LD subjects wore an opaque mask (Mindfold Relaxation Mask; Mindfold Inc.) covering both eyes. Since additional mechanisms may regulate visual cortical excitability during periods with eyes closed, we instructed observers to keep their eyes open under the mask, to better isolate the effects of deprivation. To ensure observers staying awake for the time of deprivation, they were instructed to listen to auditory media of their choice and were attended by the experimenter. Figure 2*A* provides a graphical summary of the paradigm used in experiment 1.

### Experiment 2: MD

In experiment 2, we asked how the IRFs are affected by modulation of excitability at the level of monocular processing. With this aim, we adjusted both stimulation and deprivation procedures to separate responses from left and right eye (summary of the paradigm shown in Fig. 2*B*). The stimuli consisted of two circular patches (7° in diameter) that each followed an independent luminance sequence as described above, located at 7.5° in the left and right hemifield on the horizontal meridian. Stimulation was conducted in a stereoscopic display setup that projects the images of two displays separately to the observer eyes using mirrors (Wheatstone stereoscope). Importantly, both stimulus patches were presented at the same time, but each was only visible to the eye corresponding to its hemifield, while the fixation dot was visible to both. We chose lateralized patches over (dichoptic) central stimulation because it is currently not known whether the echo response is generated before or after binocular integration. On the other side, it has been demonstrated that a dual, lateralized stimulus arrangement elicits two separate echo responses that can be modulated independently (VanRullen and MacDonald 2012; Lozano-Soldevilla and VanRullen 2019).

The displays were two Asus PG258Q LCD monitors with a resolution of 1920 × 1080 pixels and a refresh rate of 240 fps. Both screens were updated simultaneously using one split virtual screen to avoid temporal asynchronies. Total viewing distance from screen to observer (via mirror) was 40 cm. As in experiment 1, observers sat in a dark room with their head stabilized by a chinrest.

To target deprivation specifically to input from one eye, we employed the MD paradigm established previously (Lunghi et al. 2011, 2015a, 2015b). The observers wore a translucent patch over one eye that allowed luminance but only limited contrast information to reach the retina. We chose this method over a monocular opaque patch to keep total luminance roughly balanced between the eyes. While this arguably limits comparability between our two experiments, it allowed a direct interpretation of our findings based on previous studies (discussed in more detail below). The side of deprivation (left/right eye) was pseudo-randomized and balanced across observers. To assess whether possible effects of the deprivation were related to eye-dominance, we included the relationship between side of deprivation and eye-dominance (dominant deprived vs. nondominant deprived) as a between-subject factor in our analyses (7 of 16 subjects were dominant deprived).

As in experiment 1, the observers spent the time of MD in the laboratory pursuing their own activities, but with eyes open and under sufficient lighting.

### EEG Recordings

EEG data were recorded continuously during stimulation using a 64-channel active Ag/AgCl electrode system (actiCHamp amplifier module, Brain Vision LLC). Electrodes were fixed to the head with a cap and impedances brought to 5 kΩ prior to the start of recording. The signals were digitized at a sampling rate of 1 kHz using the PyCorder software (Brain Vision LLC) and stored for offline analysis.

An EyeLink 1000 system (SR Research) was used to aid eye movement and blink artifact detection in experiment 1. In experiment 2, for the same purpose, a vertical EOG was recorded through two additional electrodes positioned above and below the left eye, with a ground electrode fixed to the forehead.

### Data Analysis

All analyses were performed using MATLAB version 9.6 (R2019a), the fieldtrip EEG toolbox (Oostenveld et al. 2011) and JASP (JASP Team 2019).

### Impulse Response Functions

From the continuous EEG recording, epochs were defined based on the onset times of the stimulus patch in each trial. The first and last 500 ms of the presented 6.25 s sequences were discarded to limit analysis to the steady-state response. The resulting epochs were re-referenced to the linked mastoids, band-pass filtered between 2 Hz and the maximum frequency in the stimulus sequence (experiment 1: 60 Hz; experiment 2: 120 Hz) using a two-pass fourth-order Butterworth IIR filter, and resampled to stimulus frame rate. Trials containing gross eye movements or blinks were discarded, based on eye-tracking data (experiment 1) or EOG recordings (experiment 2).

For each trial and electrode, the EEG epochs were z-scored and cross-correlated with the presented stimulus sequence:

$$ \mathrm{IRF}(x)=\sum_t\mathrm{stim}(t)\cdot \mathrm{EEG}\left(t+x\right) $$

where *t* is the time variable of the trial in samples, and *x* is the lag between stimulus (stim) and the recorded EEG. In experiment 2, this was done for each of the two presented stimulus sequences separately, using the same EEG epochs. Cross-correlation time series were then averaged across all trials within single conditions to obtain the IRF (cf., VanRullen and MacDonald 2012).

### Time-Frequency Analysis

Amplitude and frequency measures for all response components were extracted from power spectra of the trial-averaged IRFs. For the time-resolved analyses, this was computed using wavelet analyses (Morlet wavelets; echo response: seven-cycle wavelet in steps of 10 ms, early response in experiment 2: three-cycle wavelet in steps of 5 ms). The estimation of echo-frequencies was based on the global power spectrum within the time window of interest via fast Fourier-Transform (FFT). The individual echo frequency was calculated as the center of mass of the IRF power spectrum between 8 and 12 Hz in the respective time window.

As a confirmatory analysis in experiment 1, we also calculated an alternative measure of echo response-strength that relies only on phase information. To this end, the single-trial cross-correlations were computed again using the same EEG epochs, but without amplitude information. First, the wavelet transform of each EEG epoch was calculated as before, then the power of each time-frequency bin was normalized to 1, and the signal transformed back to the time domain (using the inverse wavelet transform). After cross-correlation of the normalized EEG signal with the stimulus sequence, we then calculated the intertrial phase coherence (ITPC; Lachaux et al. 1999) between the single-trial cross-correlations as follows:

$$ {\mathrm{ITPC}}_{tf}=\frac{1}{N}\ \left|\sum_i^N{e}^{i{\theta}_{tf}}\right| $$

where *θ_tf_* denotes the instantaneous IRF phase within a single time-frequency bin in radians, and *N* is the number of trials. The resulting time-frequency spectrum provides a measure of how consistently the EEG phase couples to stimulus phase.

### Analysis of Spatial Propagation (Experiment 1)

We examined the impact of deprivation on the spatial propagation of the echo waves by analyzing their phase distributions across the scalp, following the rationale of Lozano-Soldevilla and VanRullen (2019). As this analysis relies on a high signal-to-noise ratio (SNR) of the underlying oscillation, we did not examine spatial propagation in experiment 2, where the measured echo responses were low in amplitude.

Phase angles were computed for each electrode from the Hilbert Transform of the band-pass filtered IRF (8–12 Hz). To quantify propagation speed, we calculated pairwise phase differences between neighboring electrodes along the posterior–anterior axis (pairs Oz-POz, POz-Pz, Pz-CPz, and CPz-Cz), and then averaged these differences between pairs. To assess the effects of deprivation, we subtracted the individual phase difference time courses of control and deprivation session from each other, and tested the resulting distribution against zero (CircStat, one-sample test for the mean angle; Berens 2009).

### Within-Subject Comparisons

To compare properties of the response between sessions/conditions, we extracted measures as mean values within predefined time-frequency windows and topographical regions of interest (ROIs). For the echo component, analyses were limited to the alpha range (8–12 Hz), lags between 250 and 1000 ms (experiment 1) or 750 ms (experiment 2), and electrodes POz and Pz (experiment 1) or POz only (experiment 2), based on the topography of the baseline (control) echo response. Similar boundaries and ROIs have been used in previous studies on the echoes (Chang et al. 2017; Benedetto et al. 2018).

In experiment 2, in addition to the echo component, we also examined the early part of the IRF. This was based on a previous study reporting divergent MD effects on early visual responses to each eye (Lunghi et al. 2015a). Each of the two stimulus patches in experiment 2 was positioned in the hemifield that corresponded to the eye it was presented to, with the aim of spatially separating responses from the two eyes. In accordance with this, we defined the ROIs for the early response over all occipital and parietal electrodes on the contralateral hemisphere, excluding the midline (resulting in seven pairs: O1/O2, PO3/PO4, PO7/PO8, P1/P2, P3/P4, P5/P6, and P7/P8).

## Results

### Experiment 1: LD

In this experiment, we subjected observers to 150 min of LD and compared echo responses between control and deprivation sessions.

### Echo Amplitude

**Figure 3:**
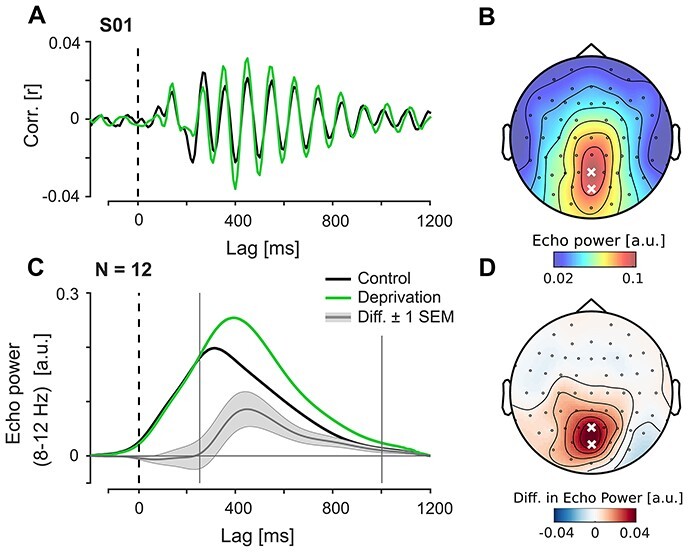
Effects of deprivation on echo amplitude (experiment 1). (*A*) Examples of IRFs measured at baseline and after LD from a single observer (electrode Pz). (*B*) Topography of echo power in the control session between lags 250–1000 ms (population average). Marked electrodes POz and Pz were used as region of interest (ROI) for the analyses on echo amplitude and frequency. (*C*) Echo envelopes (averaged over ROI) at baseline and after deprivation, and their difference. (*D*) Topography of the between-session difference in echo power between lags 250–1000 ms (population average).

As expected, we found strong echo responses in the IRF, starting at temporal lags >250 ms and persisting up to ca. 1000 ms lag in the population average (one example of an individual IRF shown in Fig. 3*A*). Representation of the IRF in the time-frequency domain revealed that the echo was centralized over posterior electrodes (Fig. 3*B*). Based on this distribution, we selected electrodes POz and Pz as our ROI for the analyses in experiment 1.

We assessed the effect of LD on echo power by computing average alpha power in the ROI between 250 and 1000 ms. This revealed a significant increase in power from control to deprivation session (paired samples *t*-test; *t*(11) = 2.673, *P* = 0.022, *d_z_* = 0.772). The postdeprivation enhancement persisted for the full echo time window but was most pronounced in the first half (Fig. 3*C*) and was topographically aligned with the peak echo response (Fig. 3*D*). Peak times of the echo envelope after deprivation were shifted by ca. 80 ms relative to the control response on average (control: 308 ms, deprivation: 391 ms), but this difference was not statistically significant (*t*(11) = 1.674, *P* = 0.122).

The main determinant of IRF power in individual frequency bands is phase-alignment between EEG and the stimulus sequence (since the response is computed from the steady-state without inclusion of the transient response to stimulus onset). However, the observed increase in echo amplitude might still have arisen from nonstimulus-specific changes in the EEG (e.g., differences in the slope of the power spectrum and within-trial variability in power, as these are not equalized by the normalization per trial). To exclude this possibility, we re-analyzed the responses from the same epochs by only including phase-information at each step: before cross-correlation, we normalized power of the EEG signal within each frequency-bin (see Material and Methods section). We then computed ITPC between the resulting single-trial cross-correlations, and averaged the results using the same boundaries as before for IRF alpha power. The between-session comparison of this measure showed the same increase after deprivation compared with control (*t*(11) = 2.323, *P* = 0.040, *d_z_* = 0.671), confirming that the observed effect was stimulus-related, and a result of increased phase coupling.

To test specifically whether nonphase-locked alpha oscillations (i.e., before cross-correlation) were also affected by LD, we extracted absolute alpha power from the raw EEG trials using the same epochs and ROI as before, and compared it between sessions. This did not reveal any significant difference (*t*(11) = 1.120, *P* = 0.287), thus providing direct support for the idea of a dissociation between alpha- and echo power within the same signal.

### Echo Frequency

**Figure 4:**
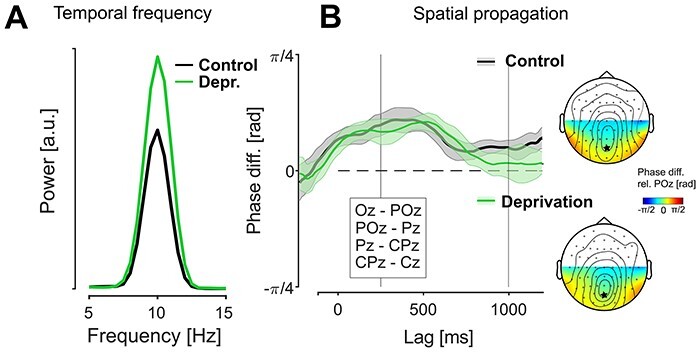
Temporal and spatial echo frequencies between sessions (experiment 1). (*A*) Global echo power spectra (lags 250–1000 ms, population average). We did not observe any systematic change in echo frequency between sessions. (*B*) Left panel: Average pairwise differences in echo phase for neighboring pairs between Oz and Cz. Shaded areas represent ±1 SEM across observers. The between-session difference showed no significant deviations from zero (*P* > 0.05, uncorrected). Right panel: Topographical distributions of echo phase over posterior electrodes (color, reference POz marked), with overlaid lines indicating the topographies of echo power in each session for reference (control: same as Fig. 2*B*). The main direction of echo propagation is from posterior to central electrodes.

Figure **4***A* shows the average global power spectra of the IRFs in the echo time window for both sessions. We quantified echo frequency for each observer as the center of mass of this spectrum between 8 and 12 Hz, using the same ROI and time window as before. The between-session comparison did not reveal any significant change in echo frequency (mean difference: 0.039 Hz; paired samples *t*-test: *t*(11) = 0.447, *P* = 0.664).

### Spatial Propagation

In addition to temporal frequency, we were also interested in a possible modulation of the spatial propagation of the echoes across the cortex. Recently, Lozano-Soldevilla and VanRullen (2019) showed that, with a central stimulation patch, the echo waves propagate mainly in a posterior-to-anterior direction, and reported an average delay of ~34 ms for the distance between Oz and Cz.

We performed a similar analysis on the echo responses from both sessions in experiment 1 and found the same pattern as Lozano-Soldevilla and VanRullen (2019). The average phase distributions in the echo time window showed a clear posterior-to-anterior gradient (Fig. **4***B*, topography plots), covering 1.676 rad between Oz and Cz on average (corresponding to ~27 ms delay). We quantified propagation speed as the average pairwise phase difference between neighboring electrodes along the midline (Fig. **4***B*; resulting in somewhat lower estimates, likely owing to inhomogeneities in the individual phase topographies). Using this measure, we found no significant difference in propagation speed between sessions when averaged over the echo time window (mean angular difference: 0.004 rad, 95% CI: [−0.113 0.122]). Post-hoc analysis revealed that the 95% CI of the difference also contained zero for all individual lags. We conclude that the deprivation had no discernible impact on echo propagation speed.

### Experiment 2: MD

In experiment 2, we investigated the differential effects of MD on separate responses originating from each eye. Previous studies have described divergent effects of MD on the early visual responses (ca. 100 ms latency) (Lunghi et al. 2015a). To relate possible changes in the echo response to these known effects, we split our analyses of the IRFs in experiment 2 in two separate time windows: the early response (0–150 ms) and the echo response (250–750 ms, see below). In both time windows, we compared responses from the control and the deprivation sessions for the nondeprived and the deprived eye.

### Early Response

**Figure 5:**
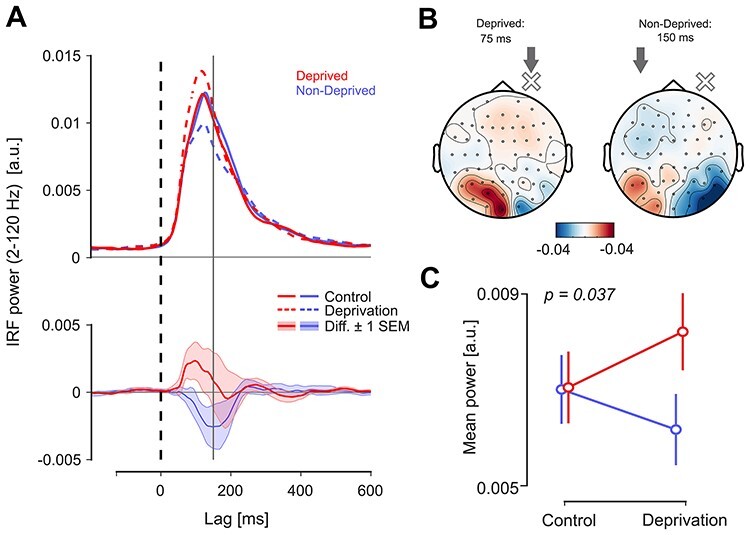
Effects of MD on the early response (experiment 2). (*A*) Top panel: Broadband (2–120 Hz) IRF power for both eyes and sessions (average over contralateral ROI and population); the time window for the early response was defined between lags 0–150 ms (vertical line). Bottom panel: Difference curves (deprivation minus control) for nondeprived and deprived eye; shaded areas represent ±1 SEM across observers. (*B*) Topography of the between-session differences in the early response. Arrows and cross mark the side of stimulation and deprivation, respectively. The topographical maps are aligned such that the deprived eye is right (note that the actual side of deprivation was pseudo-randomized). (*C*) Power of the early response in all conditions (averaged 0–150 ms), showing the significant interaction between factors SESSION and EYE (*P* = 0.037). Error bars represent ±1 SEM based on the within-subject variance.

We extracted the early response from the mean broadband power (2–120 Hz) in the contralateral ROIs (see Material and Methods section) for left- and right-eye stimulation separately, and then grouped responses according to the side of deprivation. Figure 5*A* shows the time courses of response amplitude for both eyes and sessions. The between-session comparison clearly shows a diverging pattern in response amplitude around the time of the initial peak (80–100 ms), where amplitudes were enhanced after deprivation for the deprived and attenuated for the nondeprived eye. Interestingly, modulation for the nondeprived eye reached its maximum ~150 ms lag, much later than for the deprived eye (Fig. 5*A*, bottom). As expected, both difference potentials were localized mainly over the hemisphere contralateral to stimulation (Fig. 5*B*).

We tested the overall effect by averaging power over the predefined time window (0–150 ms) and calculating a mixed-design ANOVA with the factors SESSION (control/deprivation) and EYE (nondeprived/deprived) (within-subjects), as well as DOMINANCE (dominant deprived/nondominant deprived) (between-subjects). This revealed a significant interaction SESSION × EYE (*F*(1,14) = 5.330, *P* = 0.037, η^2^_p_ = 0.276), confirming the diverging modulation of early response amplitude (Fig. 5*C*). This pattern was the same regardless whether the dominant or nondominant eye was deprived (three-way interaction term: *F*(1,14) = 0.075, *P* = 0.788, η^2^_p_ = 0.005). None of the factors exhibited a significant main effect (SESSION: *F*(1,14) = 0.124, *P* = 0.730, η^2^_p_ = 0.009; EYE: *F*(1,14) = 0.647, *P* = 0.435, η^2^_p_ = 0.044; DOMINANCE: *F*(1,14) = 2.753, *P* = 0.119, η^2^_p_ = 0.164).

### Echo Amplitude

**Figure 6:**
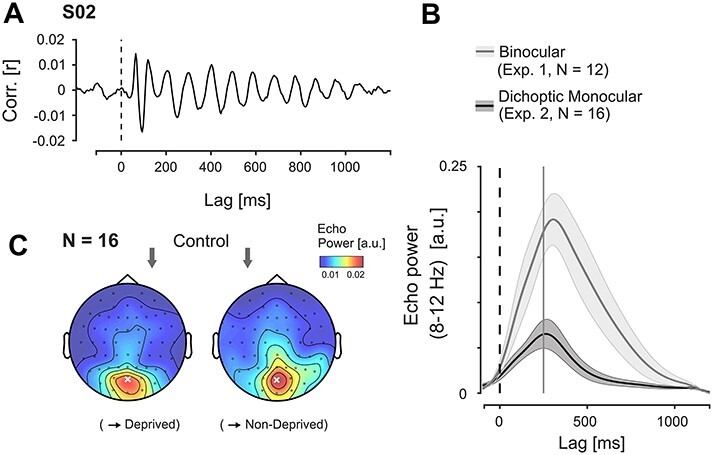
Echo responses in experiment 2. (*A*) Example of a monocular IRF in one observer (measured at electrode POz). (*B*) Comparison of echo envelopes at electrode POz, measured from binocular, single-sequence stimulation (experiment 1) and dichoptic, two-sequence stimulation in experiment 2. Based on the reduced SNR, we limited our analysis to lags 250–750 ms in experiment 2. (*C*) Topographical distribution of echo power in the control session. Electrode POz (marked x) was selected as ROI for the echo analyses in experiment 2. As before, the topographical maps are aligned such that the (designated) deprived eye is right; the arrows mark the side of stimulation.

The echo responses in experiment 2 showed the same characteristics as before (an individual example is shown in Fig. 6*A*). However, overall amplitude was greatly reduced, and amplitudes returned more quickly to the baseline level (Fig. 6*B*). Accordingly, we defined our echo time window as 250–750 ms for experiment 2.

**Figure 7:**
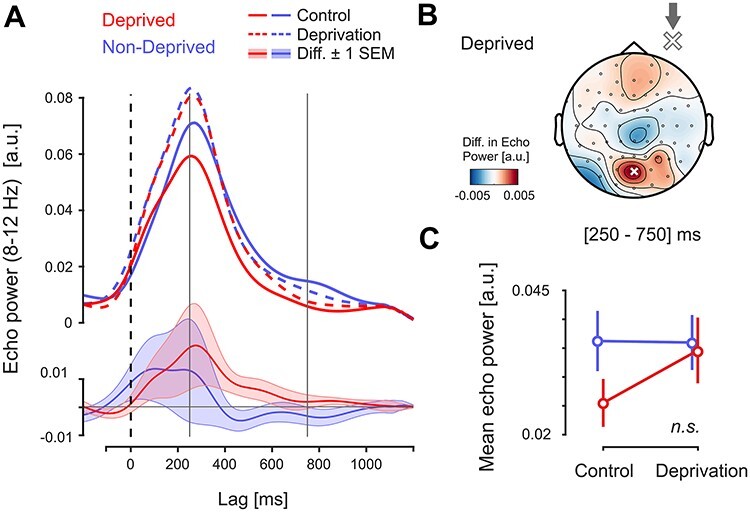
Between-session comparison of echo amplitude (experiment 2). (*A*) Top panel: Echo envelopes for both eyes and sessions (population average). Bottom panel: Difference curves (deprivation minus control) for nondeprived and deprived eye. Shaded areas represent ±1 SEM. (*B*) Topography of the between-session difference in echo amplitude for the deprived eye. As before, the topography is aligned to the side of deprivation. Note that the observed enhancement was not statistically significant. (*C*) Extracted echo power. There were no significant effects between the different conditions (interaction SESSION × EYE: *P* = 0.124; deprived eye control vs. deprivation: *P* = 0.222, corrected). Error bars represent ±1 SEM based on the within-subject variance.

The topographical representations of echo power (Fig. 6*C*) showed the expected posterior distribution for both eyes. In comparison with experiment 1, the overall distribution extended less toward central electrodes. This is consistent with our observation that the echoes generally (i.e., provided a good SNR) show a shift in power toward the center only with increasing lag.

Given the lateral positions of the stimulus patches in the visual field, we tested specifically if the echo responses were lateralized before continuing with our analysis. We averaged echo power for single conditions within each (posterior) hemisphere (using the nonoverlapping ROIs defined above for the analyses on the early response). We then calculated hemispheric differences in echo power by subtracting the ipsilateral from the contralateral side, and averaged the result between conditions to obtain a lateralization index (LI) per observer. The distribution of LI-values was not significantly greater than zero (one-tailed *t*-test: *t*(15) = 0.944, *P* = 0.180). Thus, we could not assume that the echo response was lateralized to the contralateral hemisphere. Based on the topographies of echo power in the control session (Fig. 6*C*), we selected electrode POz as our ROI for the echo analysis in experiment 2.

Figure **7**A shows the between-session comparison of echo envelopes for the two eyes. Echo power for the deprived eye showed an enhancement around the time of the peak response, persisting to a smaller extent for the full duration of the echo time window. This enhancement was topographically aligned with the overall distribution of echo power (Fig. **7***B*). The nondeprived eye showed no consistent modulation. As before, we computed a mixed-design ANOVA on the mean echo power at the ROI. This revealed that the observed modulation was not statistically significant (interaction SESSION × EYE: *F*(1,14) = 2.676, *P* = 0.124, η^2^_p_ = 0.160; three-way interaction: *F*(1,14) = 0.202, *P* = 0.660, η^2^_p_ = 0.014; main effects, SESSION: *F*(1,14) = 1.509, *P* = 0.240, η^2^_p_ = 0.097, EYE: *F*(1,14) = 0.647, *P* = 0.438, η^2^_p_ = 0.044; DOMINANCE: *F*(1,14) = 0.207, *P* = 0.656, η^2^_p_ = 0.015). Post-hoc testing showed that the selective enhancement for the deprived eye was also not significant (Tukey’s test, *P* = 0.222). The between-session differences in amplitude of early response and echo showed positive correlations at *P* < 0.1 for both eyes (deprived: *r* = 0.432, *P* = 0.096; nondeprived: *r* = 0.488, *P* = 0.057).

As a result of the overall low SNR the spatial extent of echo power across the scalp was also reduced, as compared with the echoes in experiment 1. We reasoned that this may have reduced our sensitivity in the ROI analysis, by amplifying the impact of individual differences in echo topography. To address this, we additionally compared those electrodes with maximal echo power within each condition (session/eye). As for the ROI analysis, this did not reveal any significant effects (interaction SESSION × EYE: *F*(1,14) < 0.001, *P* = 0.978, η^2^_p_ < 0.001).

### Echo Frequency

The individual echo frequencies were extracted in the same manner as for experiment 1. A mixed-design ANOVA yielded no significant interaction (SESSION × EYE: *F*(1,14) = 0.768, *P* = 0.396, η^2^_p_ = 0.052; three-way interaction *F*(1,14) = 1.802, *P* = 0.201, η^2^_p_ = 0.114), and no main effects (EYE: *F*(1,14) = 0.373, *P* = 0.551, η^2^_p_ = 0.026; SESSION: *F*(1,14) = 0.316, *P* = 0.583, η^2^_p_ = 0.022; DOMINANCE: *F*(1,14) = 0.142, *P* = 0.712, η^2^_p_ = 0.010).

## Discussion

We measured visual IRFs before and after two different regimes of visual deprivation, to examine their effects on perceptual echoes. In experiment 1, we found increased echo amplitudes following LD, while both echo frequency and spatial propagation were unaffected. In experiment 2, we targeted deprivation selectively to the input from one eye (MD). Here, early response amplitudes were modulated in opposite directions favoring the deprived eye. The echo response on the other side showed no significant modulation.

### Experiment 1: Echo Amplitude

Our goal in experiment 1 was to characterize the echoes’ dynamics under the increased visual cortical excitability that has been reported after short- and medium-term LD (Boroojerdi et al. 2000, 2001; Pitskel et al. 2007; Huang et al. 2015; Zazio et al. 2019). In this neural state, we found globally enhanced echo amplitudes relative to the baseline-state.

Our finding indicates that the echo rhythmicity is generated by a mechanism that scales with cortical excitability. This adds to existing evidence that the echoes should be viewed as independent from spontaneous alpha oscillations, which show increased synchronization instead during inhibition of visual input (Klimesch et al. 2007; Sauseng et al. 2005, 2009; Foxe and Snyder 2011), and generally when cortical excitability is low (Romei et al. 2008a, 2008b; Samaha et al. 2017). Instead, the echo may be more similar to the periodic response measured by steady-state visual evoked potentials (SSVEPs), which are enhanced by attention (Morgan et al. 1996) and following deprivation (Zhou et al. 2015). However, as SSVEPs are measured using nonrandom rhythmic stimulation, it remains unclear how a similar response would be generated from the WN sequence without synchronizing events.

Our data are also in line with the hypothesis that the echoes represent a mechanism of active sampling of visual input. On a neural level, rhythmic sampling most likely entails high-frequency spiking activity being coupled locally to the phase of alpha oscillations, for example, via phase-amplitude coupling (Canolty and Knight 2010; Jensen et al. 2014). This assumption is supported by numerous studies reporting effects in perceptual performance depending on alpha phase (see VanRullen et al. 2014; VanRullen 2016 for reviews) and even, indirectly, the phase of the echo itself (Gulbinaite et al. 2017). In this context, the increase in rhythmicity that we observed here could be explained by decreased thresholds for the spiking activity that is nested in the alpha cycle. However, if the echo propagates as a traveling wave as suggested by Lozano-Soldevilla and VanRullen (2019), the enhancement may be related mostly to spontaneous activity evoked by the echo at remote cortical locations. To assess the significance of changes in echo amplitude, it would be interesting to see how the processing of secondary stimuli at these locations is affected.

### Experiment 1: Echo Frequency

We expected to find a shift in echo frequency following deprivation, more specifically toward higher frequencies, based on previous reports that cortical excitability increases peak alpha frequency (Mierau et al. 2017), and evidence that the echo frequency itself varies adaptively (Benedetto et al. 2018). It is remarkable that LD did not evoke a similar frequency shift, in particular when considering its effect on echo amplitude.

From a functional perspective, our result supports the view that echo frequency is actively controlled to match up with “visual demand.” Benedetto et al. (2018) targeted this specifically by presenting stimuli with either high or low ambient luminance. They found that the echoes in the low-luminance condition were increased in frequency, and interpreted this as an adaptive change of the sampling mechanism to account for reduced stimulus reliability. Similar adaptive shifts have been reported for nonstimulus-locked alpha in a temporal integration/segregation task (Wutz et al. 2018). Conversely, our stimuli were designed to be identical between sessions, to isolate the effect of neural state. Echo frequency may be less prone to this type of “passive” manipulation, or even actively compensate for it. The question how echo frequency depends differently on extrinsic versus intrinsic manipulation may be an interesting direction for future studies.

Our analysis on the spatial propagation of the echoes was exploratory. It is still unknown over what cortical distance and through which mechanism the echoes propagate. Our aim was to illuminate this by analyzing the relationship of temporal frequency and propagation speed. Among other aspects, the co-variation between these two parameters could give indication as to whether the echo propagates via coupled oscillators (Ermentrout and Kleinfeld 2001; Zhang et al. 2018). Thus, future studies on the dynamics of echo frequency should investigate it in parallel with spatial propagation.

### Experiment 2: Effects of MD

As expected, we found a divergent modulation of early responses between deprived (enhancement) and nondeprived eye (attenuation) after MD. This matches the pattern Lunghi et al. (2015a) found after the same duration of MD by examining ERP components. The replication of this effect confirms the efficacy of our MD regime and extends the previous finding. Specifically, while Lunghi et al. measured responses to transient stimuli, the IRFs are derived from continuous stimulation, excluding the transient stimulus-onset response. Thus, the imbalance in response amplitude between the eyes persists in the steady-state response. This is consistent with increased SSVEP amplitudes found in the deprived eye after MD (Zhou et al. 2015) and the sustained perceptual shifts under binocular rivalry (Lunghi et al. 2011; Zhou et al. 2013).

The echoes in experiment 2 did not show the same modulation as the early response. The between-session comparison showed a small enhancement for the deprived eye that was aligned with the peak response. However, this effect was not statistically significant, and the non-deprived eye showed no changes.

While these results present a complex pattern, it can be concluded that echo amplitude was not modulated by MD to the extent of the effects for the early response, or the enhancement of echo amplitude after LD. There are several possible explanations for the lack of an effect of MD on the echoes. Most importantly, the results in experiment 2 overall indicate that the echoes do not provide an input-specific stimulus representation: The echo responses from monocular stimulation were low in SNR compared with binocular viewing conditions. A reason for this may be that the echo is generated in V1 after binocular integration, meaning that the integrated response is attenuated by the input from the eye that is presented with black background. The echo responses also showed no lateralization in amplitude to the contralateral hemisphere. This suggests that the echoes are a global response that takes place across V1 (and possibly beyond), in line with the assumption that they propagate as traveling waves away from the retinotopic representation of the stimulus patch (Lozano-Soldevilla and VanRullen 2019). This distributed response pattern might make the echo largely insensitive to monocular modulation.

From a functional perspective, this explanation seems plausible: while the echoes’ temporal periodicity presumably serves the sampling of continuous input (VanRullen 2016), their spatial propagation may coordinate this sampling across retinotopic coordinates (Lozano-Soldevilla and VanRullen 2019). This coordination should incorporate the full (binocular) visual field and be affected by low-level modulation only on a global level.

Of course, it is possible that the observed enhancement for the deprived eye merely failed to reach statistical significance due to the reduced SNR. A selective enhancement would have similar implications as our results from experiment 1. In line with the explanation presented above, this effect may be limited to the first cycles of the echo in closer proximity to the retinotopic representation.

It should be considered here as an additional limitation that the two experimental sessions were recorded on two different days. Given the low SNR of the echoes in experiment 2, natural fluctuations in echo amplitude across different days may have been too large in relation to possible smaller effects of the deprivation to be detected. We consider this inevitable, however, because the alternative of having both experimental sessions on a given day could have introduced other confounds, for example, time of the day or alertness.

Another explanation for our results is that the echoes were unaffected specifically by the translucent patch (contrast deprivation). We chose this method to match with the standard MD regime in the recent literature (Lunghi et al. 2011, 2015a, 2015b; Zhou et al. 2013, 2015; Binda et al. 2018). Indeed, there is reason to assume that the effects of opaque and translucent patching are very similar: both induce states of reduced inhibition and increase gain of the deprived input in V1 (enhanced BOLD responses; opaque: Boroojerdi et al. 2000; translucent: Binda et al. 2018) and a direct comparison of the two methods in MD showed no differences in perceptual effects (Zhou et al. 2013). On the other hand, the nature of the stimuli in our study is not specific to these effects. In their study, Binda et al. (2018) found that the effects of translucent patching are exerted primarily through the parvocellular pathway, consistent with the loss of high spatial frequency information during deprivation. The echoes are measured in response to a luminance-modulated stimulus without spatial features, and may therefore be more strongly affected by opaque than translucent patching. However, we argue that this is unlikely the main reason for the lack of an effect of MD on the echoes, most importantly because the same stimuli showed an effect for the early response.

As noted above, the two paradigms in this study (LD and MD) were designed mainly to match with previous studies reporting effects of deprivation and the stimulation adjusted to best isolate putative effects on the echo responses. A side-by-side comparison of the results from the two experiments has to take several differences in procedure into account (most importantly binocular vs. monocular stimulation, but also central vs. lateral stimulation, one vs. two patches). Further studies will be needed to establish in particular how the perceptual echoes differ between monocular and binocular viewing conditions, in order to better understand how they are generated on a cortical level.

## Conclusion

This study characterizes the dynamics of perceptual echoes following a disruption in the excitation/inhibition balance. Our data show that the echoes’ synchrony scales with cortical excitability, linking the response to active visual processing as opposed to inhibitory modulation. This is in line with the previous studies that have demonstrated that the echoes may represent a mechanism of rhythmic sampling (VanRullen 2016; Benedetto et al. 2018), that is, the transformation of a continuous visual input stream into periodically structured activity. Conversely, the present results provide further insight into how this may be achieved on a cortical level. Specifically, between the two experiments, our data suggest that the echoes are more susceptible to global modulation and are likely generated after binocular integration. This may be tentatively seen as support for the assumption that the echoes propagate as traveling waves across large regions of visual cortex.

## References

[ref1] Ahveninen J , Lin FH, Kivisaari R, Autti T, Hämäläinen M, Stufflebeam S, Belliveau JW, Kähkönen S. 2007. MRI-constrained spectral imaging of benzodiazepine modulation of spontaneous neuromagnetic activity in human cortex. Neuroimage. 35:577–582. 17300962 10.1016/j.neuroimage.2006.12.033

[ref2] Benedetto A , Lozano-Soldevilla D, VanRullen R. 2018. Different responses of spontaneous and stimulus-related alpha activity to ambient luminance changes. Eur J Neurosci. 48:2599–2608. 29205618 10.1111/ejn.13791

[ref3] Berens P. 2009. CircStat: a MATLAB toolbox for circular statistics. J Stat Softw. 31:186637.

[ref4] Binda P , Kurzawski JW, Lunghi C, Biagi L, Tosetti M, Morrone MC. 2018. Response to short-term deprivation of the human adult visual cortex measured with 7T bold. Elife. 7:1–25. 10.7554/eLife.40014 PMC6298775 30475210

[ref5] Boroojerdi B , Battaglia F, Muellbacher W, Cohen LG. 2001. Mechanisms underlying rapid experience-dependent plasticity in the human visual cortex. Proc Natl Acad Sci U S A. 98:14698–14701. 11734655 10.1073/pnas.251357198 PMC64744

[ref6] Boroojerdi B , Bushara KO, Corwell B, Immisch I, Battaglia F, Muellbacher W, Cohen LG. 2000. Enhanced excitability of the human visual cortex induced by short-term light deprivation. Cereb Cortex. 10:529–534. 10847602 10.1093/cercor/10.5.529

[ref7] Brainard DH . 1997. The psychophysics toolbox. Spat Vis. 10:433–436. 9176952

[ref8] Canolty RT , Knight RT. 2010. The functional role of cross-frequency coupling. Trends Cogn Sci. 14:506–515. 20932795 10.1016/j.tics.2010.09.001 PMC3359652

[ref9] Chang AY-C , Schwartzman DJ, VanRullen R, Kanai R, Seth AK. 2017. Visual perceptual echo reflects learning of regularities in rapid luminance sequences. J Neurosci. 37:14–16. 10.1523/JNEUROSCI.3714-16.2017 PMC6596871 28765331

[ref10] Doppelmayr M , Klimesch W, Pachinger T, Ripper B. 1998. The functional significance of absolute power with respect to event-related desynchronization. Brain Topogr. 11:133–140. 9880171 10.1023/a:1022206622348

[ref11] Ermentrout GB , Kleinfeld D. 2001. Traveling electrical waves in cortex: insights from phase dynamics and speculation on a computational role. Neuron. 29:33–44. 11182079 10.1016/s0896-6273(01)00178-7

[ref12] Fierro B , Brighina F, Vitello G, Piazza A, Scalia S, Giglia G, Daniele O, Pascual-Leone A. 2005. Modulatory effects of low- and high-frequency repetitive transcranial magnetic stimulation on visual cortex of healthy subjects undergoing light deprivation. J Physiol. 565:659–665. 15760946 10.1113/jphysiol.2004.080184 PMC1464536

[ref13] Foxe JJ , Snyder AC. 2011. The role of alpha-band brain oscillations as a sensory suppression mechanism during selective attention. Front Psychol. 2:1–13. 21779269 10.3389/fpsyg.2011.00154 PMC3132683

[ref14] Gaillard C , Ben Hadj Hassen S, Di Bello F, Bihan-Poudec Y, VanRullen R, Ben HS. 2020. Prefrontal attentional saccades explore space rhythmically. Nat Commun. 11:925. 32066740 10.1038/s41467-020-14649-7 PMC7026397

[ref15] Gulbinaite R , İlhan B, VanRullen R. 2017. The triple-flash illusion reveals a driving role of alpha-band reverberations in visual perception. J Neurosci. 37:7219–7230. 28663196 10.1523/JNEUROSCI.3929-16.2017 PMC6705726

[ref16] Hindriks R , van Putten MJAM. 2013. Thalamo-cortical mechanisms underlying changes in amplitude and frequency of human alpha oscillations. Neuroimage. 70:150–163. 23266701 10.1016/j.neuroimage.2012.12.018

[ref17] Huang S , Hokenson K, Bandyopadhyay S, Russek SJ, Kirkwood A. 2015. Brief dark exposure reduces tonic inhibition in visual cortex. J Neurosci. 35:15916–15920. 26631472 10.1523/JNEUROSCI.1813-15.2015 PMC4666916

[ref18] JASP Team . 2019. JASP (Version 0.10.2).

[ref19] Jensen O. 2002. Oscillations in the alpha band (9-12 Hz) increase with memory load during retention in a short-term memory task. Cereb Cortex. 12:877–882. 12122036 10.1093/cercor/12.8.877

[ref20] Jensen O , Gips B, Bergmann TO, Bonnefond M. 2014. Temporal coding organized by coupled alpha and gamma oscillations prioritize visual processing. Trends Neurosci. 37:357–369. 24836381 10.1016/j.tins.2014.04.001

[ref21] Klimesch W , Sauseng P, Hanslmayr S. 2007. EEG alpha oscillations: the inhibition-timing hypothesis. Brain Res Rev. 53:63–88. 16887192 10.1016/j.brainresrev.2006.06.003

[ref22] Lachaux JP , Rodriguez E, Martinerie J, Varela FJ. 1999. Measuring phase synchrony in brain signals. Hum Brain Mapp. 8:194–208. 10619414 10.1002/(SICI)1097-0193(1999)8:4<194::AID-HBM4>3.0.CO;2-C PMC6873296

[ref23] Lagerlund TD , Sharbrough FW. 1989. Computer simulation of the generation of the electroencephalogram. Electroencephalogr Clin Neurophysiol. 72:31–40. 2464473 10.1016/0013-4694(89)90028-x

[ref24] Lopes Da Silva FH , van Lierop THMT, Schrijer CF, van Leeuwen W. 1973. Organization of Thalamic and Cortical Alpha Rhythms: spectra and coherences. Electroencephalogr Clin Neurophysiol. 35:627–639. 4128158 10.1016/0013-4694(73)90216-2

[ref25] Lozano-Soldevilla D , Ter Huurne N, Cools R, Jensen O. 2014. GABAergic modulation of visual gamma and alpha oscillations and its consequences for working memory performance. Curr Biol. 24:2878–2887. 25454585 10.1016/j.cub.2014.10.017

[ref26] Lozano-Soldevilla D , VanRullen R. 2019. The hidden spatial dimension of alpha: 10-Hz perceptual echoes propagate as periodic traveling waves in the human brain. Cell Rep. 26:374–380. 30625320 10.1016/j.celrep.2018.12.058 PMC6326161

[ref27] Lunghi C , Berchicci M, Morrone MC, Di Russo F. 2015a. Short-term monocular deprivation alters early components of visual evoked potentials. J Physiol. 593:4361–4372. 26119530 10.1113/JP270950 PMC4594246

[ref28] Lunghi C , Burr DC, Morrone MC. 2011. Brief periods of monocular deprivation disrupt ocular balance in human adult visual cortex. Curr Biol. 21:R538–R539. 21783029 10.1016/j.cub.2011.06.004

[ref29] Lunghi C , Emir UE, Morrone MC, Bridge H. 2015b. Short-term monocular deprivation alters GABA in the adult human visual cortex. Curr Biol. 25:1496–1501. 26004760 10.1016/j.cub.2015.04.021 PMC5040500

[ref30] Mierau A , Klimesch W, Lefebvre J. 2017. State-dependent alpha peak frequency shifts: experimental evidence, potential mechanisms and functional implications. Neuroscience. 360:146–154. 28739525 10.1016/j.neuroscience.2017.07.037

[ref31] Morgan ST , Hansen JC, Hillyard SA, Posner M. 1996. Selective attention to stimulus location modulates the steady-state visual evoked potential. Proc Natl Acad Sci U S A. 93:4770–4774. 8643478 10.1073/pnas.93.10.4770 PMC39354

[ref32] Oostenveld R , Fries P, Maris E, Schoffelen JM. 2011. FieldTrip: open source software for advanced analysis of MEG, EEG, and invasive electrophysiological data. Comput Intell Neurosci. 2011:156869. 21253357 10.1155/2011/156869 PMC3021840

[ref33] Pitskel NB , Merabet LB, Ramos-Estebanez C, Kauffman T, Pascual-Leone A. 2007. Time-dependent changes in cortical excitability after prolonged visual deprivation. Neuroreport. 18:1703–1707. 17921872 10.1097/WNR.0b013e3282f0d2c1

[ref34] Romei V , Brodbeck V, Michel C, Amedi A, Pascual-Leone A, Thut G. 2008a. Spontaneous fluctuations in posterior alpha-band EEG activity reflect variability in excitability of human visual areas. Cereb Cortex. 18:2010–2018. 18093905 10.1093/cercor/bhm229 PMC2517102

[ref35] Romei V , Rihs T, Brodbeck V, Thut G. 2008b. Resting electroencephalogram alpha-power over posterior sites indexes baseline visual cortex excitability. Neuroreport. 19:203–208. 18185109 10.1097/WNR.0b013e3282f454c4

[ref36] Samaha J , Gosseries O, Postle BR. 2017. Distinct oscillatory frequencies underlie excitability of human occipital and parietal cortex. J Neurosci. 37:2824–2833. 28179556 10.1523/JNEUROSCI.3413-16.2017 PMC5354329

[ref37] Sauseng P , Klimesch W, Heise KF, Gruber WR, Holz E, Karim AA, Glennon M, Gerloff C, Birbaumer N, Hummel FC. 2009. Brain oscillatory substrates of visual short-term memory capacity. Curr Biol. 19:1846–1852. 19913428 10.1016/j.cub.2009.08.062

[ref38] Sauseng P , Klimesch W, Stadler W, Schabus M, Doppelmayr M, Hanslmayr S, Gruber WR, Birbaumer N. 2005. A shift of visual spatial attention is selectively associated with human EEG alpha activity. Eur J Neurosci. 22:2917–2926. 16324126 10.1111/j.1460-9568.2005.04482.x

[ref39] VanRullen R. 2016. Perceptual Cycles. Trends Cogn Sci. 20:723–735. 27567317 10.1016/j.tics.2016.07.006

[ref40] VanRullen R , MacDonald JSP. 2012. Perceptual echoes at 10 Hz in the human brain. Curr Biol. 22:995–999. 22560609 10.1016/j.cub.2012.03.050

[ref41] VanRullen R , Zoefel B, Ilhan B. 2014. On the cyclic nature of perception in vision versus audition. Philos Trans R Soc B Biol Sci. 369:20130214. 10.1098/rstb.2013.0214 PMC3965168 24639585

[ref42] Wutz A , Melcher D, Samaha J. 2018. Frequency modulation of neural oscillations according to visual task demands. Proc Natl Acad Sci U S A. 115:1346–1351. 29358390 10.1073/pnas.1713318115 PMC5819398

[ref43] Zazio A , Bortoletto M, Ruzzoli M, Miniussi C, Veniero D. 2019. Perceptual and physiological consequences of dark adaptation: a TMS-EEG study. Brain Topogr. 32 (5):773–782. 31076949 10.1007/s10548-019-00715-x

[ref44] Zhang H , Watrous AJ, Patel A, Jacobs J. 2018. Theta and alpha oscillations are traveling waves in the human neocortex. Neuron. 98:1269–1281.e4. 29887341 10.1016/j.neuron.2018.05.019 PMC6534129

[ref45] Zhou J , Baker DH, Simard M, Saint-Amour D, Hess RF. 2015. Short-term monocular patching boosts the patched eye’s response in visual cortex. Restor Neurol Neurosci. 33:381–387. 26410580 10.3233/RNN-140472 PMC4923712

[ref46] Zhou J , Clavagnier S, Hess RF. 2013. Short-term monocular deprivation strengthens the patched eye’s contribution to binocular combination. J Vis. 13:1–10. 10.1167/13.5.12 23599416

